# *Pseudomonas aeruginosa* Biofilm Response and Resistance to Cold Atmospheric Pressure Plasma Is Linked to the Redox-Active Molecule Phenazine

**DOI:** 10.1371/journal.pone.0130373

**Published:** 2015-06-26

**Authors:** Anne Mai-Prochnow, Mark Bradbury, Kostya Ostrikov, Anthony B. Murphy

**Affiliations:** 1 CSIRO Manufacturing Flagship, P.O. Box 218, Lindfield, NSW 2070, Australia; 2 CSIRO Food and Nutrition Flagship, 11 Julius Ave, North Ryde, NSW 2113, Australia; 3 Institute for Health and Biomedical Innovation, School of Chemistry, Physics and Earth Sciences, Queensland University of Technology, Brisbane, QLD 4000, Australia; University Paul Sabatier, FRANCE

## Abstract

*Pseudomonas aeruginosa* is an important opportunistic pathogen displaying high antibiotic resistance. Its resistance is in part due to its outstanding ability to form biofilms on a range of biotic and abiotic surfaces leading to difficult-to-treat, often long-term infections. Cold atmospheric plasma (CAP) is a new, promising antibacterial treatment to combat antibiotic-resistant bacteria. Plasma is ionized gas that has antibacterial properties through the generation of a mix of reactive oxygen and nitrogen species (RONS), excited molecules, charged particles and UV photons. Our results show the efficient removal of *P*. *aeruginosa* biofilms using a plasma jet (kINPen med), with no viable cells detected after 5 min treatment and no attached biofilm cells visible with confocal microscopy after 10 min plasma treatment. Because of its multi-factorial action, it is widely presumed that the development of bacterial resistance to plasma is unlikely. However, our results indicate that a short plasma treatment (3 min) may lead to the emergence of a small number of surviving cells exhibiting enhanced resistance to subsequent plasma exposure. Interestingly, these cells also exhibited a higher degree of resistance to hydrogen peroxide. Whole genome comparison between surviving cells and control cells revealed 10 distinct polymorphic regions, including four belonging to the redox active, antibiotic pigment phenazine. Subsequently, the interaction between phenazine production and CAP resistance was demonstrated in biofilms of transposon mutants disrupted in different phenazine pathway genes which exhibited significantly altered sensitivity to CAP.

## Introduction

Antibiotics and other conventional antimicrobial methods often fail to eradicate disease causing bacteria due to an ever-increasing development of resistance from bacteria [1,2]. This resistance is even higher when cells live in a biofilm community compared to planktonic, single-cell lifestyle [3]. Biofilms are responsible for the majority of human infections as well as a range of problems in industry, including fouling of pipelines and ship hulls [4]. Biofilm cells live in a multilayer community, enclosed by an extracellular matrix rather than individual free swimming cells. The extracellular matrix is composed of polymeric substance, DNA, proteins and polysaccharides [5]. Cells in a biofilm can communicate via signal molecules that diffuse through the biofilm. Biofilm cells are known to be in a different physiological state than their planktonic counterparts. This state, together with the protective extracellular matrix, gives them greater resistance to antibiotics, chemical disinfection (e.g. hydrogen peroxide) and physical removal [3].

*Pseudomonas aeruginosa* is an opportunistic pathogen with a particularly high rate of antibiotic resistance, in part due to its potent ability to form biofilms [6,7]. It is responsible for many hospital-acquired infections, in particular of chronic wounds [8], and represents a major problem for patients with cystic fibrosis [9]. *P*. *aeruginosa* produces several virulence factors. One of them is the redox active phenazine compound pyocyanin [10]. Phenazines produced by fluorescent pseudomonads are broad range, biologically-active metabolites with functions in virulence [11,12]. Their antibiotic activity is mostly due to the ability to undergo redox-cycling in the presence of various reducing agents, leading to the accumulation of toxic levels of superoxide and hydrogen peroxide [13]. Hassan et al. showed that the toxic action of pyocyanin is dependent on oxygen and diminished in the presence of superoxide dismutase and catalase [13]. Moreover, phenazines were shown to be involved in *P*. *aeruginosa* induced killing of *Caenorhabditis elegans* via the generation of reactive oxygen species [12] They also play an important role in co-infections of *P*. *aeruginosa* and *Candida albicans* in lungs of cystic fibrosis patient, where ethanol released from the fungus alters the spectrum of antifungal phenazines [14]. However, there is also some evidence that phenazines have a protective role in oxidative stress regulation in *P*. *aeruginosa*. Vinckx et al. [15] showed that the transcriptional regulator Oxyr is important for defence against oxidative stress in *P*. *aeruginosa*. Interestingly, it was demonstrated that a Δ*oxyR* mutant has increased pyocyanin levels. Moreover, while the Δ*oxyR* mutant is almost unable to grow in media where no pyocyanin is produced, the addition of external pyocyanin can restore growth ability to normal wild-type levels, suggesting a protective action for this compound [15]. Another study suggests that phenazines can confer an advantage for *P*. *aeruginosa* biofilm growth. Because of their redox potential, phenazines can act as an electron shuttle between bacteria and external substrates, extending the possible depth of respiration for oxygen-deprived cells inside microcolonies [16]. Moreover, it was shown that phenazines are important for colony morphology on agar plates and biofilm microcolonies. A wrinkly colony morphology in the absence of phenazines was suggested to be an adaptation to maximize oxygen accessibility in case of a redox imbalance [17].

Cold atmospheric plasma (CAP) has attracted attention as a useful tool to eradicate bacteria [18]. Plasma, or ionized gas, is the fourth state of matter. CAP, as the name suggests, is formed at atmospheric pressure, and the molecular, atomic, radical and ionic species are at close to room temperature. CAP can be generated by a range of devices using different gases (most commonly argon, helium, nitrogen, air or oxygen) or gas mixtures. Through the generation of a reactive mix of atoms, excited molecules, charged particles, reactive oxygen species (ROS) and reactive nitrogen species (RNS), and UV photons, CAP has been shown to inactivate microorganisms, including bacteria and their spores [19,20].

Several studies have proposed different plasma-proposed mechanisms as being responsible for the killing of bacteria. The antibacterial modes of actions vary depending on the type of plasma generated [21]. Three main plasma-induced mechanisms of bacterial cell damage are hypothesized: a) permeabilisation of the cell wall and/or membrane, b) protein damage through reactive oxygen and nitrogen species (RONS) and c) UV damage of DNA (reviewed in [18]). For example, a study by Joshi et al. [22] demonstrated that ROS are directly responsible for membrane lipid peroxidation and DNA damage in *E*. *coli* during the application of CAP. Using bacteriophage MS2, Alshraiedeh et al. showed that phage inactivation increased with increasing oxygen content of the plasma, also indicating a role for ROS in plasma activated killing [23]. Interestingly, Gram negative cells have been reported to be more susceptible to plasma damage, suggesting that cell wall architecture is important for inactivation [24].

CAP has often been shown to kill planktonic cells, but only a few studies have used biofilms to demonstrate its effectiveness [25–28]. Biofilms have been reported to be more protected from plasma killing than planktonic cells [29,30], with the level of protection dependant on the biofilm’s thickness [24]. A study on *Chromobacterium violaceum* showed a different survival curve for biofilm cells compared to planktonic cells, and suggests a more complex inactivation mechanism [27]. Similarly, a rapid initial killing followed by a slower decline in cell numbers was observed for *P*. *aeruginosa* biofilms [26]. The slower, later decline in viable cells was proposed to be due to the presence of an exopolysaccharide biofilm matrix or due to a shielding effect from initial cell debris [25]. In addition to biofilm-mediated protection from plasma killing, the polysaccharide polymer dextran was found to have a protective role in the lactic acid bacterium *Weissella confusa*, indicating strain-specific protection mechanisms [30].

In order to remain effective antibacterial agents need to evade bacterial defense systems and avoid inducing resistance mechanisms. Bacteria can be intrinsically resistant to antibacterial agents or acquire a resistance, for example through mutations [31,32]. A repeated selection pressure from frequent exposure can aggravate the occurrence of acquired resistance, as it has been seen with the overuse of antibiotics [2]. Moreover, the generation of persister cells, dormant variants of regular cells with high antibiotic resistance, may exacerbate the problem. It is hypothesized that persister cell formation may be activated by stress responses and is particularly common during the biofilm mode of growth [33]. It was recently shown that *Acinetobacter baumannii* increased the formation of persister cells when grown in the presence of pyocyanin-producing *P*. *aeruginosa*. The increased persister cell formation has been proposed to be a protective mechanism against pyocyanin-induced oxidative stress [34].

Several studies demonstrate that CAP has the potential for use as an effective antimicrobial tool. However, as with other antimicrobial measures, the question of possible resistance to CAP needs to be addressed. This is particularly important for microbial biofilms, as these are known to commonly have a higher resistance to antimicrobials. The pathogen *P*. *aeruginosa* has a number of resistance mechanisms, including beta-lactamase, upregulated efflux pumps and mutations leading to alterations in antibiotic target molecules, as well as the ability to generate dormant persister cells. Our objective was to investigate whether resistance to CAP treatment occurs in *P*. *aeruginosa* biofilms as well as a possible resistance mechanism. We chose *P*. *aeruginosa* colonies that had survived low doses of CAP treatment for further investigations. Biofilms of these strains were subjected to additional rounds of CAP treatment to show a possible higher resistance. To investigate a resistance mechanism the chosen colonies were subjected to whole genome sequencing and several mutations, including in phenazine biosynthesis genes were identified. To further demonstrate the involvement of phenazine in *P*. *aeruginosa* resistance to CAP, biofilms of phenazine transposon mutants were tested for altered sensitivity to plasma. These results have important implications for the use of CAP in biofilm settings.

## Materials and Methods

### Bacterial Strains

Bacteria were cultivated on nutrient agar (1g l^-1^ `Lab-Lemco’ powder, 2g l^-1^ yeast extract, 5g l^-1^ peptone, 5g l^-1^ sodium chloride, 15g l^-1^ agar, pH 7.4; Oxoid) using standard methods (Table 1). Overnight cultures were inoculated into 10 ml nutrient broth and incubated at 37°C, 150 rpm shaking.

**Table 1:** Bacterial strains used in this study.

Strain	Description	Reference
***P*. *aeruginosa*** ATCC9027	Wild-type	Food Research Ryde Bacteriology Culture Collection
***P*. *aeruginosa*** GC1	Variant treated 5 min argon gas	This study
***P*. *aeruginosa*** GC2	Variant treated 5 min argon gas	This study
***P*. *aeruginosa*** 2P1	Variant treated 2 min argon plasma	This study
***P*. *aeruginosa*** 2P2	Variant treated 2 min argon plasma	This study
***P*. *aeruginosa*** 5P1	Variant treated 5 min argon plasma	This study
***P*. *aeruginosa*** 5P2	Variant treated 5 min argon plasma	This study
***P*. *aeruginosa* MPAO1 WT**	Wild-type	B. Iglewski, [35]
***P*. *aeruginosa*** PW4333	Strain, phzD2-E04::ISlacZ/hah disrupted in phenazine biosynthesis protein D2	[35]
***P*. *aeruginosa*** PW4335	Strain phzE2-C11::ISlacZ/hah disrupted in phenazine biosynthesis protein E2	[35]

### Biofilms

Biofilms were established in a CDC (Centre for Disease Control and Prevention) biofilm reactor on 24 individual glass or stainless steel coupons of 1.27 cm diameter and 0.3 cm thick [36]. Briefly, 1 ml of an overnight culture (OD = 1) was inoculated into the reactor, containing 500 ml of tryptic soy broth TSB (600 mg l^-1^) and incubated for 24 h stirring (120 rpm) at 22°C without flow. After 24 h the flow was turned on and sterile 100 mg TSB l^-1^ was continuously pumped through the reactor (flow rate 11.7 ml min^-1^) for another 24 h. Before treatment, coupons were aseptically removed and the underside of the coupons swabbed with ethanol to remove any bacteria not designated for treatment. Coupons were then placed bacteria side up into empty Petri dishes for treatment.

### Plasma treatment

**Fig 1:**
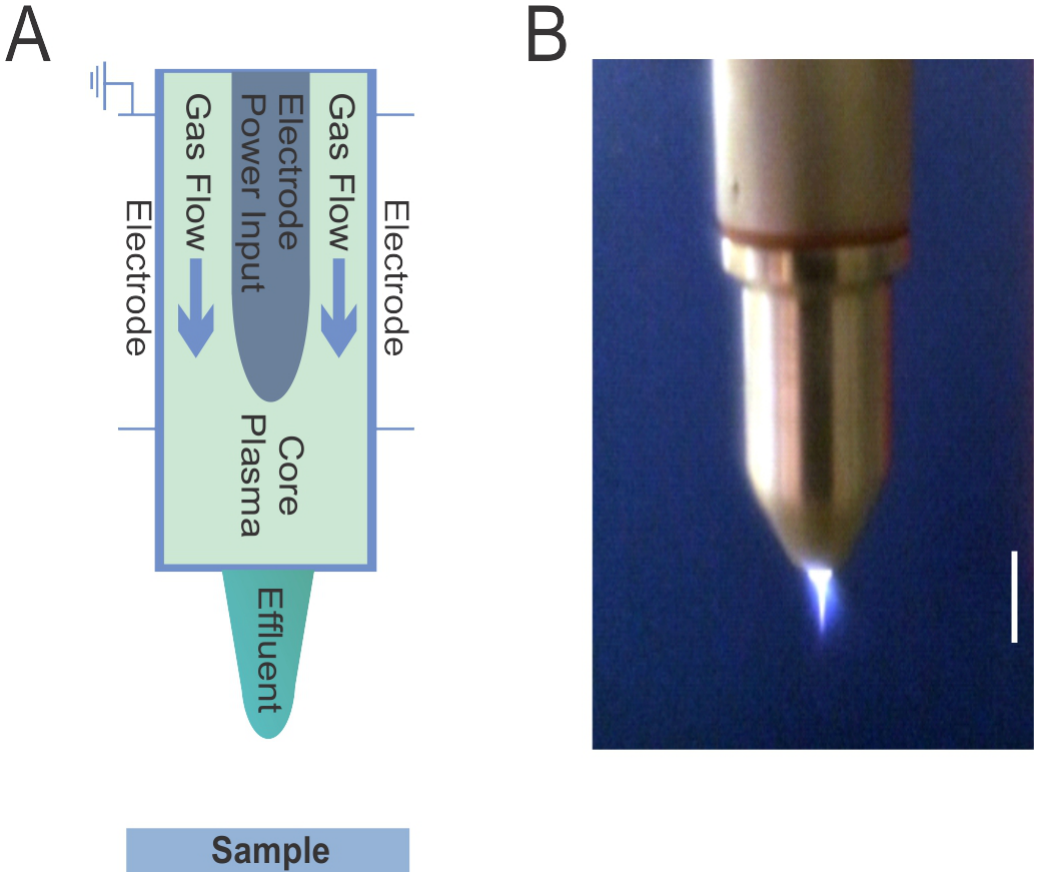
Plasma use in biomedical settings. Schematic diagram of the plasma source kINPen med (A) and photograph of plasma jet operation (B). The white bar is 1 cm.

Plasma treatment was performed using the kINPen med (Neoplas tools GmbH, Greifswald, Germany) [37]. The kINPen hand-held nozzle was connected to a base unit with a gas feeding bottle. Argon was used as a feeding gas and plasma pulses generated at a frequency of 1.82 MHz. The visible plasma effluent was approximately 1 cm long and 1 mm in diameter (Fig 1). The gas flow rate was set to 4.2 slm (7.1 Pa m^3^ s^-1^). The distance to the sample surface was 1 cm. Plasma treatment was conducted in triplicate at 1, 3, 5 and 10 min, respectively. As a control, a 10 min gas treatment without igniting plasma was performed at 3.9 slm (6.6 Pa m^3^ s^-1^). A slightly lower gas rate for the control was necessary to prevent the kINPen from automatically igniting the plasma at >4.0 slm.

To exclude a possible heating effect from the plasma, the temperature was measured using a negative temperature coefficient (NTC) thermistor (10K/3435). The sensor was placed at the coupon location and the temperature recorded over 10 minutes.

After treatment coupons were placed into 3 ml sterile phosphate buffered saline (PBS). Coupon surfaces were scraped with a sterile spatula to remove cells. In addition coupons were sonicated for 1 min to dissolve possible cell clumps. The removal of cells from coupons was confirmed by microscopy (data not shown). Following serial dilution, cells were plated onto nutrient agar and incubated for 24 h at 37°C, followed by another 2 days at room temperature before counting colony forming units (CFU).

### Microscopy

Following plasma treatment biofilms were stained with the BacLight Live/dead stain containing the 2 stock solutions green Syto 9 and red propidium iodide (Molecular Probes, Eugene, Oregon, USA) according to the manufacturer’s instruction and fixed with 10% para-formaldehyde. Biofilms were visualized using the Olympus FV1000 confocal laser scanning microscope. Five positions were randomly chosen from the centre of the coupon and a complete scan through the biofilm was performed. The biofilm thickness was estimated from the vertical images using the scale bar function from the manufacturer’s image analysis software (Olympus Fluoview version 3.1a).

### Plasma resistance test

**Fig 2:**
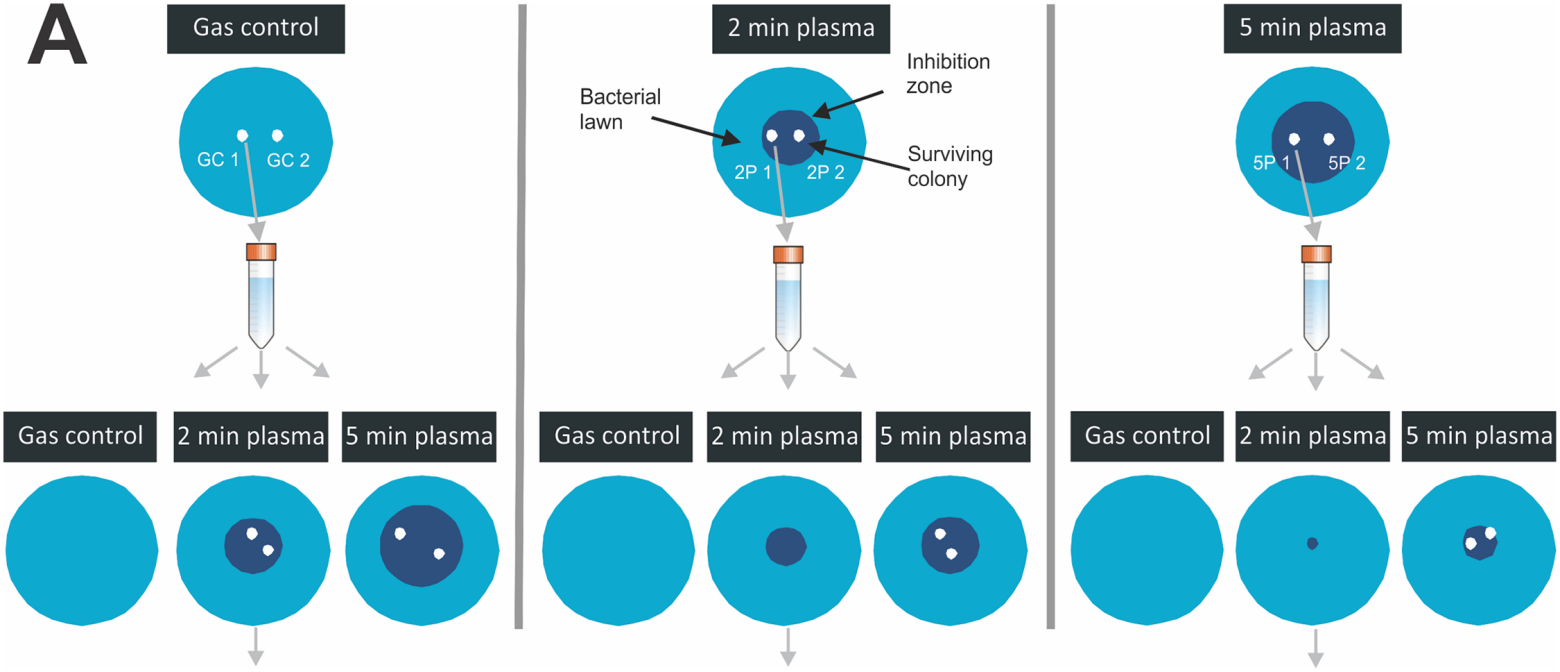
Schematic of experimental set-up to isolate plasma resistant colonies. Colonies were picked from agar plates after initial 5 min argon gas control treatment, initial 2 min argon plasma treatment and initial 5 min plasma treatment, respectively.

To investigate a possible resistance of *P*. *aeruginosa* to plasma treatment, initially an agar-plate-based method was employed, where isolates from within the zone of inhibition after an initial plasma exposure were exposed to a second plasma treatment. Briefly, 100 μl of overnight culture was spread onto nutrient agar plates and dried for 1 h before argon plasma treatment (2 or 5 min) or 5 min argon gas treatment in the centre of the agar plate. Plates were then incubated for 24 h and the size of clearing zone noted. Surviving colonies were picked from the clearing zone, sub-cultured and used to prepare fresh lawns in triplicates. Each of the triplicate plates was then again subjected to gas, 2 min plasma or 5 min plasma treatment. The size of inhibition zone was used as an indicator of plasma-induced inactivation. The experimental set-up is shown in Fig 2.

### Hydrogen peroxide resistance

The 5 isolated variants were inoculated overnight in 5 ml NB before diluting 20x in micro titre plates. The range of hydrogen peroxide concentrations tested was between 5 and 100 mM. Plates were incubated at 30°C and read every 15 min at 600 nm for 48 h in Bioscreen C (Thermo Fisher Scientific).

### Illumina Sequencing and variant discovery

Bacterial colonies growing in the plasma inhibition zone were chosen for whole genome DNA sequencing. Two colonies were picked from 2 min plasma treatment plate (2P1, 2P2), two colonies from the 5 min plasma treatment plate (5P1, 5P2) and 1 colony from the control, gas treatment plate (GC1). Colonies were isolated with a sterile toothpick, inoculated into fresh nutrient broth and incubated for 14 h at 37°C to reach stationary phase. DNA was extracted using DNeasy Blood and Tissue kit (Qiagen) and purified using Wizard SV Gel and PCR clean-up system (Promega). DNA sequencing was performed at Ramaciotti Centre for Genomics (UNSW, Australia) using Illumina MiSeq technology.

Reads generated were aligned against a reference *de novo* assembly of the GC1 genome. The reference draft GC1 genome was assembled using Velvet v1.0.0 [38] implemented on a local Galaxy server [39]. Default settings were used with a hash length of 75 bp. Error correction of reads was performed prior to assembly using Trimmomatic v0.30 (http://www.usadellab.org/cms/index.php?page=trimmomatic).

Sequencing reads generated from the 2P1, 2P2, 5P1 and 5P2 were aligned against the reference assembly using Geneious (v7.0.6); http://www.geneious.com/) using the map to reference function. Variant discovery between the gas-control isolates and plasma-treated isolates was performed using the find variations/SNPs function. Default SNP search parameters were used with a minimum variant frequency of 0.6. Individual variants identified were then manually checked and the potential gene product identified by using blastN against the non-redundant nucleotide collection. Raw sequencing reads have been submitted to NCBI short read archive (SRA) under the BioProject number PRJNA269551 (http://www.ncbi.nlm.nih.gov/bioproject/PRJNA269551). Sequencing reads are located at ftp://ftp-trace.ncbi.nlm.nih.gov/sra/sra-instant/reads/ByRun/sra/SRR/SRR204/SRR2043986/ (2 min plasma treatment); ftp://ftp-trace.ncbi.nlm.nih.gov/sra/sra-instant/reads/ByRun/sra/SRR/SRR204/SRR2043987/ (5 min plasma treatment) and ftp://ftp-trace.ncbi.nlm.nih.gov/sra/sra-instant/reads/ByRun/sra/SRR/SRR204/SRR2043045/ (gas control).

### Biofilm formation and plasma treatment of *phz* mutants

To investigate a possible role of phenazine in resistance to CAP, transposon mutants disrupted in *phzD* (strain PW4333) and *phzE* (strain PW4335) were used. Mutant strains along with the corresponding wild-type (MPAO1) were acquired from a two-allele library from the nonprofit cost centre at the University of Washington (Table 1).

Biofilms were allowed to form by placing a coupon into 1 ml nutrient broth with 5 μl overnight culture at 37°C for 24 h. Before plasma treatment 50 μl of 2.4 μM pyocyanin (Sapphire Bioscience, Waterloo, Australia) in PBS and 10% ethanol was added directly to the coupons. As a control PBS and 10% ethanol was used. A 3 min argon plasma treatment followed by CFU counts was performed as described above.

## Results

### Biofilm removal by atmospheric pressure plasma

Plasma generated with the kINPen med (Fig 1) was used to treat the antibiotic-resistant, biofilm-forming strain *Pseudomonas aeruginosa* (ATCC9027) grown on stainless steel coupons.

**Fig 3:**
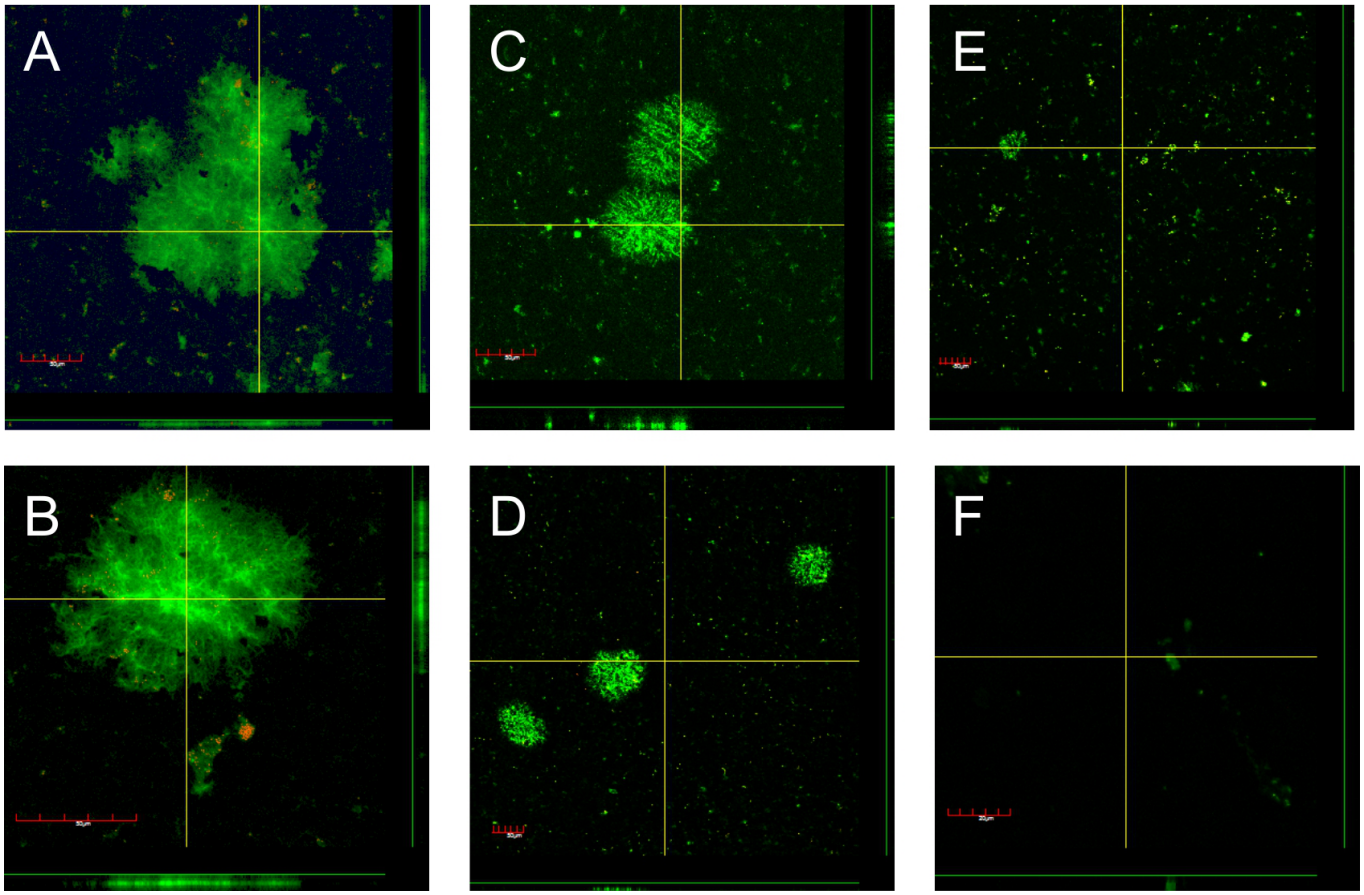
Confocal images of *P*. *aeruginosa* biofilms cells after argon plasma treatment stained with *Bac*Light Live/Dead. Viable cells are stained green and dead cells are stained red A) Untreated control, B) 10 min argon gas control, C) 1 min plasma, D) 3 min plasma, E) 5 min plasma and F) 10 min plasma treatment. Each image shows a representative horizontal section (main picture), and two vertical sections (to the right of and below the green lines on the right-hand side and bottom of the main picture, respectively). The vertical sections correspond to the two yellow lines in the main picture.

After a range of treatment times, biofilms were subjected to viable cell counts (Table 2) and confocal microscopy (Fig 3) to investigate the effect of CAP on biofilms. The untreated control biofilms reached cell numbers of 1.3 x 10^6^ CFU per coupon (Table 2). The gas control showed a slight decrease in CFU numbers (1.2 x 10^5^ per coupon), possibly due to a drying effect from the gas flow. Plasma treatments after 1 and 3 minutes produced similar results as the gas control with a reduction of viable cell numbers by only 1 log compared to the control. However, no viable cells were detected after 5 min of plasma treatment (Table 2).

**Table 2:** CFU counts of *P*. *aeruginosa* biofilms cells after argon plasma treatment.

Treatment	CFU counts (per coupon)
Untreated control	1.3 (± 0.2) x 106
Gas control	1.2 (± 0.2) x 10^5^
1 min CAP	5.4 (± 0.9) x 10^4^
3 min CAP	6.4 (± 0.1) x 10^4^
5 min CAP	*
10 min CAP	*

* indicates counts were below detection limit (1 x 10^2^)

In addition to CFU numbers, a second set of biofilms grown on glass coupons was observed with confocal laser scanning microscopy. The untreated control showed attached, viable cells forming microcolonies approximately 15 μm thick (Fig 3A). The gas control showed similar size microcolonies. A few single dead cells were observed in the gas control, presumably due to a drying effect from the gas flow. After only 1 min of plasma treatment, microcolonies were significantly smaller and some dead cells appeared. Large microcolonies are removed after only 3 minutes of plasma treatment with most of the biofilm removed after 5 min and no cells left after 10 min (Fig 3).

Monitoring of the temperature of the plasma revealed only a marginal increase over the time-course of treatment. The temperature was 31.4°C after 3 min, 31.8°C after 5 min and 32.6°C after 10 min treatment time, respectively, showing that cell killing was not due to high temperature.

### Resistance to plasma

We found that longer plasma treatment (5 min) leads to larger inhibition zones on agar plates, similarly to an antibiotic concentration disk test. However, within the plasma inhibition zone, several surviving colonies formed after incubation of the agar plate, prompting us to investigate a possible resistance of these colonies to plasma. Interestingly, after isolating these colonies (that survived the first round plasma treatment), a higher resistance to subsequent plasma exposure was noted. This was evident from a smaller zone of inhibition compared to the control sample, which had only been exposed to argon gas before being treated with plasma (data not shown).

### Resistance to hydrogen peroxide

**Fig 4:**
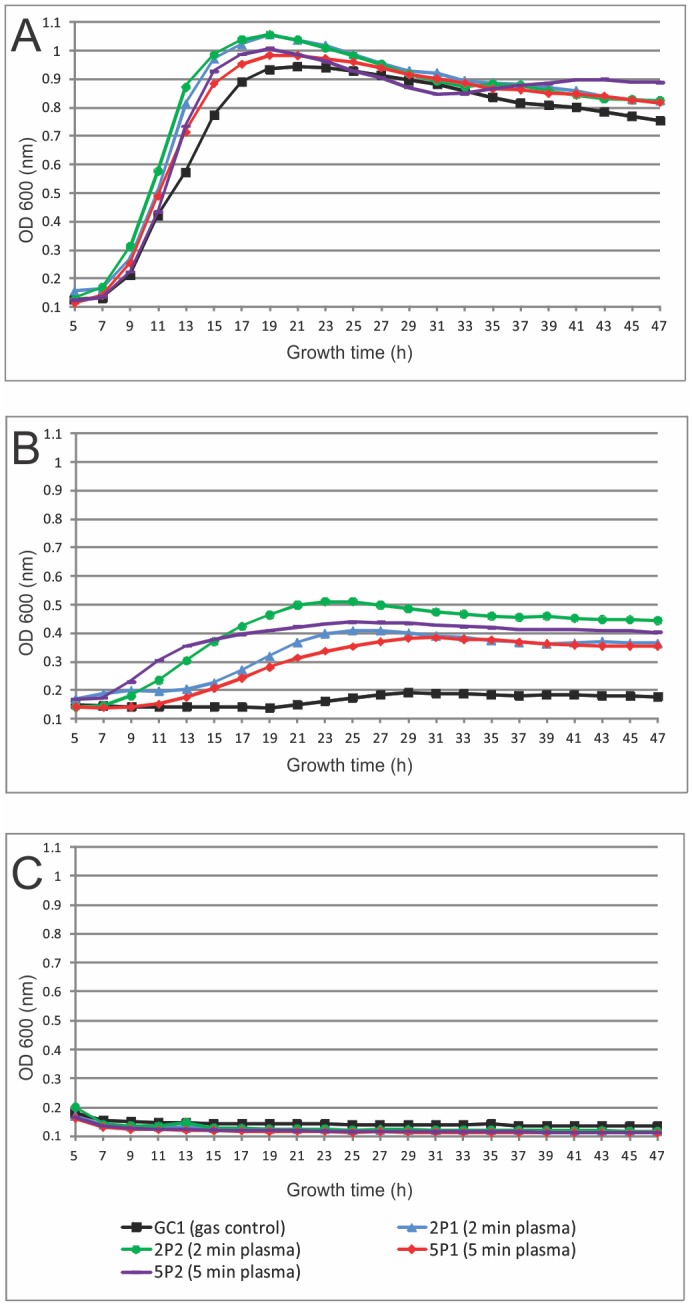
Growth curve of plasma resistant colonies. Cells were exposed to A) 5 mM H_2_O_2_, B) 50 mM H_2_O_2_ and C) 100 mM H_2_O_2_.

Isolated colonies that survived initial plasma treatment of 2 or 5 min, respectively were tested for their ability to grow in hydrogen peroxide solutions of different concentrations. Five variants were chosen, including 2 variants from a plate exposed to 2 min argon plasma (2P1, 2P2), 2 variants from a plate exposed to 5 min argon plasma (5P1, 5P2) and 1 variant from a gas control plate (GC1). At a concentration of 5 mM H_2_O_2_, all variants had a similar growth rate and reached a similar final optical density (Fig 4A). However, at a 50 mM H_2_O_2_ concentration, colonies that were isolated as survivors from a CAP treatment plate were found to grow significantly better than the variant isolated from a gas treated plate (Fig 4B). Strain 2P2 reached the highest final optical density (OD), which was about 2.5 times higher than the final OD from the control strain. Of the four plasma surviving strains (2P1, 2P2, 5P1 and 5P2), strain 5P1 reached the lowest final OD and also had the most delayed lag time when exposed to 50 mM H_2_O_2_. Interestingly the growth curves of strains 2P1 and 5P1 are more similar than the 2P1 and 2P2 growth curves. All strains are completely inhibited to grow at 100 mM H_2_O_2_ concentration (Fig 4C).

### Identifying mutations in plasma resistant colonies

In order to further characterize the plasma-resistant colonies, whole genome DNA sequencing was carried out on the 5 variants. A single nucleotide polymorphism (SNP) comparison between variants was performed against the genome assembly of the control isolate. Putative gene products encoded by these polymorphic regions were identified by using blastN. The SNP analysis indicated 10 distinct polymorphisms (6 SNP transitions, 3 SNP transversions and 1 substitution) in 7 different genes or intergenic regions in plasma-treated variants, including AAA transcriptional regulator, diaminopimelate decarboxylase, conjugal transfer protein, pyridoxamine 5-phosphate oxidase, and the phenazine biosynthesis proteins PhzE and PhzD (Table 3).

**Table 3:** Polymorphisms in plasma treated *P*. *aeruginosa* variants.

Gene Product	Variant	Change	Position	Polymorphism type
Probable pyridoxamine 5-phosphate oxidase followed by **phenazine** biosynthesis protein	**5P2**	C -> T	6,349,784	SNP (transition)
**Phenazine** biosynthesis protein PhzE	**2P1, 2P2**	A -> G	6,351,727	SNP (transition)
**Phenazine** biosynthesis protein PhzD	**2P2, 5P1, 5P2**	T -> C	6,353,019	SNP (transition)
	**2P1, 2P2, 5P1, 5P2**	T -> C	6,353,125	SNP (transition)
Putative transcriptional regulator (AAA domain protein)	**2P1, 2P2**	C -> T	1,197,847	SNP (transition)
Diaminopimelate decarboxylase	**2P2**	T -> A	2,157,427	SNP (transversion)
Conjugal transfer protein	**2P1**	G -> C	2,945,334	SNP (transversion)
	**2P1, 2P2**	G -> A	2,945,346	SNP (transition)
	**2P1, 2P2**	GT -> AC	2,945,350	Substitution
Intergenic region between 16S RNA gene and putative membrane protein	**5P1**	T -> G	5,174,776	SNP (transversion)

(2P1, 2P2 = recovered after 2 min plasma treatment; 5P1, 5P2 = recovered after 5 min plasma treatment)

Interestingly several of the same SNPs occur in more than one of the variants and the same SNP in phenazine D protein occurs in all 4 variants sequenced. This SNP changes the AA Asparagine to Serine.

### Phenazine plays a role in plasma resistance in *P*. *aeruginosa*

**Fig 5:**
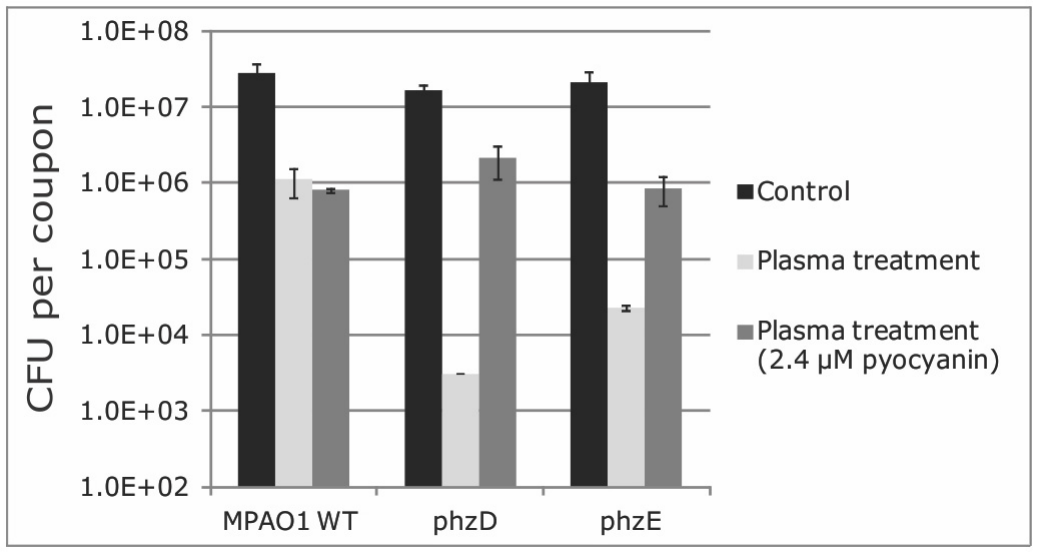
Survival of PAO1 phenazine mutants upon plasma exposure. CFU counts per coupon of MPAO1 wild-type and *phz* mutant biofilms exposed to 3 min argon plasma. Error bars denote standard deviation of triplicate cell counts.

Because 4 of the 10 detected DNA changes upon plasma exposure relate to phenazine biosynthesis genes (*phzD* and *phzE*), we further investigated their role in plasma resistance of *P*. *aeruginosa*. Biofilms of transposon mutants *phzD* (strain PW4333) and Δ*phzE* (strain PW4335) were allowed to form on stainless steel coupons and then treated with CAP for 3 min before performing CFU counts. Fig 5 shows that the mutants are significantly more sensitive to CAP treatment. A 3 min CAP treatment of wild-type biofilms led to a 1.5 log reduction compared to a 3.5 and 3.0 log reduction for the *phz* mutant biofilms, respectively. Add back of the phenazine pyocyanin to the *phz* mutant biofilms restored CAP induced killing to wild-type levels, suggesting that phenazine plays a role in protecting *P*. *aeruginosa* from argon plasma (Fig 5).

## Discussion

Pathogenic bacteria exhibiting resistance to antibiotic agents have become an area of great concern. Whilst antibiotic resistance in bacteria occurs naturally to some extent [1,40], inappropriate use and overuse of these drugs has increased this process at an alarming rate [2]. In particular biofilms, which account for over 60% of infections, show a high degree of resistance [3]. This has several causes, including altered metabolic state, presence of an extracellular matrix, oxidative stress response, differential gene or protein expression, and the presence of persister cells [33,41].

Results of our study clearly demonstrate that *Pseudomonas aeruginosa* biofilms can be eradicated using a 10 min plasma treatment with a kINPen med. Other studies have shown plasma-mediated killing of biofilm cells using different plasma sources [25,27,42,43], but a complete removal was only observed in one other study [44]. A study by Xiong et al. confirmed that plasma is able to penetrate *Porphyromonas gingivalis* biofilms of 15 μm thickness [45]. In another study, a hand-held plasma pen was shown to inactivate a 25.5 μm thick biofilm of *Enterococcus faecalis* [28] and Chen et al. also suggests a penetration depth of plasma chemistry of 10–50 μm into a biofilm [46]. Interestingly, in our study no culturable cells were detected after 5 min plasma treatment, when there were still some Syto 9 stained cells visible at the same time-point using confocal microscopy. The discordance between culturable cells and visible cells under the microscope may be that not all cells were efficiently scraped off from the coupon before cell counting. Moreover, it is feasible that green fluorescent cells visible with confocal microscopy are not viable despite staining with Syto 9 and thus do not grow on agar plates, leading to a lower CFU number compared to visible fluorescent cells. It has been previously described that plasma treatment may lead to cells entering the viable but non-culturable state (VBNC) [47,48]. This state is characterized by cells having an intact membrane, respiration activity, gene transcription and protein synthesis, but not being able to form colonies on culture media in laboratory conditions [49].

The mode of action of plasma-mediated bacterial killing has been suggested to be a combination of direct permeabilisation of the cell wall, damage of intracellular proteins from reactive oxygen and nitrogen species and direct chemical alteration of DNA [50,51]. For example, oxidative species generated by CAP (OH and NO) have a direct effect on both the membrane and cell wall of microorganisms via generation of an oxidative stress response. This can lead to lipid peroxidation, inactivation of Fe-S-dependent dehydratases, inactivation of mononuclear iron proteins and DNA damage, which ultimately cause detrimental oxidative cell damage [18,22,50]. Because of its multi-factorial mode of action, the chance of bacteria developing resistance to plasma treatment is often considered to be low. However, our data demonstrate that a short plasma treatment may contribute to surviving cells exhibiting enhanced resistance to subsequent plasma treatment.

Bacteria can have a natural resistance or an acquired resistance (through mutations or acquiring resistance genes) to antibacterial agents [52]. Zimmermann [52] performed a study to investigate a possible build-up of natural and acquired resistance of *E*. *coli* to CAP. The study showed that no natural resistance to CAP occurs in the tested *E*. *coli* population, and no build-up for secondary resistance was observed [52]. In our study, 2 scenarios can be hypothesized. It is possible that colonies grew in the plasma treatment zone after incubation because they either a) had a favorable natural mutation before the treatment or b) plasma exposure induced such a mutation. It is feasible that the experimental approach (choosing surviving colonies after incubation) led to enrichment of plasma-resistant persister cells.

The second scenario, that plasma exposure can induce mutations, could occur via stimulating a stress response. By producing a reactive mix of species, CAP is a DNA-damaging agent that could have the effect of inducing a stress response and subsequent mutations that lead to plasma resistance and the formation of persister cells. *P*. *aeruginosa* can adapt to changing environments (such as oxidative stress) with beneficial mutations. For example, MutS and DinB (DNA Polymerase IV) have been shown to be involved in DNA-damaged mutagenesis [53]. DNA-damaging RONS were shown to accumulate in the centre of *P*. *aeruginosa* microcolonies, leading to cell death [54]. Furthermore microcolonies are hot spots for accumulation of mutations [55]. Conibear et al showed that up to 100-fold higher mutation rate occurs in biofilms compared to planktonic growth in *P*. *aeruginosa*. This mutator phenotype can enhance microcolony-based growth and is proposed to be important for a better adaptation to growth in overcrowded conditions [55]. There is also evidence that an accumulation of RONS in *P*. *aeruginosa* biofilms leads to DNA lesions in phage which in turn triggers the occurrence of phenotypic variants [56]. It is hypothesized that creating many phenotypic variants generates genetic diversity, which in turn secures survival under different environmental conditions, including oxidative stress [57].

Our results show that 4 of the 10 detected DNA changes upon plasma exposure relate to phenazine biosynthesis genes. Phenazines are brightly colored small molecules naturally produced by Pseudomonas. *P*. *aeruginosa* contains a complex phenazine biosynthetic pathway consisting of two homologous core loci (phzA1B1C1D1E1F1G1 and phzA2B2C2D2E2F2G2) [58]. Phenazines themselves are a potent broad-spectrum antibiotic, giving Pseudomonads an advantage that allows them to outcompete other bacteria [59]. Furthermore, phenazine were shown to increase resistance to antibiotics [34] and promote anaerobic survival via extracellular electron transfer [60]. There is evidence that phenazines have a protective role in oxidative stress regulation [15]. In *P*. *aeruginosa* OxyR regulates the defense to oxidative stress and *oxyR* mutation affects production of the phenazine redox-active molecule pyocyanin. OxyR mutants can only grow in Pseudomonas P agar, which induces pyocyanin, but cannot grow well in Luria Bertani media unless external pyocyanin is added [15] demonstrating that the phenazine molecule pyocyanin has a protective role in *P*. *aeruginosa*.

Our results suggest a role for phenazines in the bacterial response to plasma exposure as biofilms of different *phz* transposon mutants are more sensitive to CAP treatment. In addition our results show that variants that survived CAP treatment could also grow under higher concentrations of hydrogen peroxide compared to gas treated cells. This clearly demonstrates a role for oxidative stress in plasma-mediated killing. Moreover, a link between oxidative stress regulons (OxyR, SoxR and OspR) and different phenazine productions has been reported [15,61,62] supporting our results for the involvement of phenazines in plasma survival and resistance.

In conclusion, our study demonstrates that CAP can be a valuable tool in combating bacterial biofilms. However, care must be taken as low doses may lead to the emergence of resistant bacteria and the formation of persister cells.
